# Inpatient care 50 years after the process of deinstitutionalisation

**DOI:** 10.1007/s00127-013-0788-6

**Published:** 2013-11-09

**Authors:** Emese Csipke, Clare Flach, Paul McCrone, Diana Rose, Jacqueline Tilley, Til Wykes, Tom Craig

**Affiliations:** 1 Department of Psychology, Institute of Psychiatry, King’s College London, 16 De Crespigny Park, London, SE5 8AF UK; 2 Social Psychiatry, Health Service and Population Research Department, Institute of Psychiatry, King’s College London, London, UK; 3 Health Service and Population Research Department, Centre for the Economics of Mental and Physical Health, Institute of Psychiatry, King’s College London, London, UK; 4 Service User Research Enterprise, Health Service and Population Research Department, Institute of Psychiatry, King’s College London, London, UK

**Keywords:** Acute, Inpatient, Psychiatric, Service users, Activities

## Abstract

**Purpose:**

Throughout the past 50 years mental health services have aimed to provide and improve high quality inpatient care. It is not clear whether there has been improvement as service users and nursing staff have both expressed frustration at the lack of therapeutic activities. In particular, it may be that the changing levels of symptoms over the past 50 years may affect engagement with ward activities.

**Methods:**

Eight wards in a health care trust in London serving an inner city and urban populations participated. Data were collected on participation in activities and 116 service users’ perceptions of acute care as well as clinical factors.

**Results:**

Less time was spent participating in activities today than 50 years ago, while one quarter of service users reported taking part in no activities at all. Uptake of activities was related to more positive service user perceptions of the wards. Symptom severity did not impact the frequency of participation in activities, although those who took part in no activities at all had higher negative symptoms scores.

**Conclusions:**

Service users’ uptake of activities was not related to the severity of their illness. This belies the belief that the acutely ill cannot take part in meaningful activities. This study supports the view that more therapeutic activities could be taken up by the acutely ill and are in fact appreciated.

## Introduction

Growing concerns about the poor living conditions in asylums and changing attitudes toward mental illness and institutionalisation have led to the gradual transition from the long-term hospitalisation of those with mental health problems to an increase in community treatment. However, hospitalisation for acute episodes is still an indispensable feature of mental health care and service users continue to complain about the quality of inpatient care [1]. Around 50 years ago John Wing and George Brown started the seminal ‘Three Hospitals’ study that demonstrated that hospitals which provided richer social environments and social opportunities had markedly less disturbance in verbal and social behaviour, as well as patients with the fewest negative symptoms [2]. Between 1960 and 1964 the poverty of the social environment lessened in all three hospitals and simultaneously the clinical condition of patients improved (specifically a reduction in negative symptoms). In particular, those patients on wards whose social environments were enriched improved clinically with the best predictive factor being a reduced amount of time spent doing nothing. Those patients with the least social interaction, the least access to the outside world, with the fewest interesting activities to take part in and who spent the majority of their time doing nothing were the most unwell, but as their environments became more stimulating they too showed some improvements.

Although much has changed in the nature of inpatient provision with the move away from the hospital asylum, the essential association between social interaction and better clinical outcome continued to be reported across the next two decades [3] as has the low level of patient and staff interaction [4]. It seems likely that although no recent study has examined the issue, these associations remain. Bed numbers have continued to fall and wards are generally now reserved for the most acutely ill with consequent increases in the proportion of inpatients who are compulsorily detained and increases in levels of behavioural disturbance [5–7]. In turn, this fraught environment can lead to difficulties in staff recruitment and retention. Taken together this often brings about ward environments that appear to be more custodial than therapeutic. A MIND report [1] examining acute wards across England and Wales found that service users often complained about the intense boredom they experienced. Service users recognised that the lack of therapeutic activities (or the cancellation of those on offer) is often due to staff shortages and/or too many demands being placed on staff. It been reported that nurses blamed the need to focus on resolving crises for a small number of patients, as well as the increase in administrative duties expected of them, as the primary reasons for not being able to spend time on therapeutic activities or in direct patient contact [4, 8]. Although the majority of research in this area has originated in the UK, a recent review of the literature on patient activities and interaction encompassed international perspective and found that this lack of service user engagement seems to hold true across North American, Australia and Europe [9].

A Sainsbury Centre for Mental Health [10] report provided a more upbeat picture, finding that 73 % of ward managers across England reported that both practical activities (e.g. cooking skills) and talking therapies occurred regularly on their wards, and 64 % reported leisure activities (e.g. film nights) occurring regularly. Specifically, therapies with a strong evidence base, such as CBT, only occurred on 20 % of the wards. The recommendations include increasing the availability of training in therapeutic activities which have an evidence base [10]. What is omitted from the report is any discussion that, although activities of all types may be something the wards aim to provide and indeed make plans to do so, how often they happen in practice may be a different matter.

## Aims of the study

We investigate the hypothesis that levels of activity are associated with clinical functioning just as they were 50 years ago and that activity and one-to-one contact continue to be closely correlated with service users’ satisfaction with inpatient care.

## Method

### Design

The present study is a cross-sectional exploration of the activities on inpatient wards that was gathered as baseline data preparatory to an inpatient activity intervention in a large Trust serving both inner city and suburban communities. A detailed description of the whole programme of research is given on www.perceive.iop.kcl.ac.uk. Data for the current paper were collected over a 1-month period for each of eight acute inpatient wards.

All service users who were on the ward for at least 7 days, spoke English and were able to consent were eligible from wards providing all acute care for a defined geographical area involving both inner city and inner urban areas. Ward staff introduced the researchers to service users individually, or at ward community meetings. After gaining informed consent, assessments were carried out in ward offices or other private rooms on the ward with just the researcher and service user present, as approved by the Bromley Ethics Committee.

### Measures

#### Clinical information on patients

1. Global assessment of functioning [11] is a 100-point rating scale of a patient’s overall level functioning that is based on observation, interview and medical records. The total score and subscores for symptoms (GAF-S) and functioning (GAF-F) were examined.
2. Positive and negative syndrome scale: PANSS [12] a 30-item instrument that evaluates the presence, absence and severity of positive (e.g. hallucinations and delusions), negative (e.g. blunted affect and passive social withdrawal) symptoms and general psychopathology (e.g. anxiety, depression). Researchers were trained in how to conduct the interviews and were subsequently approved by a senior member of the research team (TC). Researchers practiced and several tapes were listened to until they reached the point that there was no disagreement with any rating by more than one point and never between the critical 3/4 distinction.
3. Nurses’ observational scale for inpatient evaluation: NOSIE [13], a 12-item scale designed for nurses largely focused on the assessment of socially unacceptable behaviour. Nurses are asked to rate participants on items such as ‘Sits, unless directed into activity’.
4. Client services receipt inventory-inpatient; CITRINE [14] gathers data on the care contacts and therapeutic activities of patients whilst on a psychiatric ward, which can then be used for costing of inpatient psychiatric services. The instrument is designed to be completed in a 5–10 min interview with a service user and asks about the number of activities and number and duration of staff contacts in the week prior to the interview. A week was chosen as the assessment window since during the development of the measure it was found that people who are acutely unwell have difficulty remembering their activities for a longer period accurately.
5. Views on inpatient care: VOICE [15] is a 19-item, self-report measure of service users’ perceptions of acute inpatient care. It was developed by service users using a participatory methodology, has strong psychometric properties and is acceptable to inpatients. It also has high internal consistency (α = 0.92) and the test–retest reliability (*n* = 40) was high (*ρ* = 0.88, CI 0.81–0.95). An example of an item is ‘Staff give me medication instead of talking to me’.

#### Ward level data

Information regarding the number and grade of staff on duty, the time nurses spent in one-on-one observations with service users and the percentage service users under a section of the Mental Health Act constitutes the ward level data. The number of incidents of self-harm, suicide attempt, violence and aggression was also recorded over a 30-day period.

### Statistical analysis

Data were analysed using SPSS version 18 and STATA v.10. Simple descriptive statistics were used to examine differences in numbers of group activities and one-to-one contacts by service user demographic characteristics, the inpatient setting and the associations of these with clinical measures of symptoms (PANSS, GAF), nurse observations (NOSIE) and service user views (VOICE). Statistically significant bivariate associations were further explored using generalised estimating equations to fit a poisson regression model with the frequency spent in either group activity or one-to-one time as the dependent variable. As patients on the same ward were likely to be more similar in terms of exposure to group and one-to-one contacts these data were treated as clustered at ward level.

## Results

### The wards investigated

Eight single-sex wards (4 male and 4 female) were included in this analysis. With the exception of a ward-in-the community that had a specific remit to provide crisis care for women, all were typical of hospital-based inpatient care. The ward in the community falls under the remit of inpatient services in the trust so it was, therefore, included. Ward sizes range from 8 to 25 beds with an overall average of 18 beds per ward. Staffing levels do not vary greatly by ward with, on average 0.3 (SD 0.03) staff per bed during the day (approximately 4–6 staff on duty, 2 staff on smallest ward). An additional 1.3 staff per ward are employed from 9 am to 5 pm. There are slightly fewer staff on the wards at night, around 0.2 (SD 0.04) staff per bed. A total of 257 patients were admitted to these wards over a 30-day period.

There were nine violent incidents (four on one ward), and three incidents involving self-harm (one on each of three wards). There was no significant difference between wards in terms of participants’ acuity (PANSS, GAF-S), level of functioning (GAF-F, NOISIE) or satisfaction with care between the wards.

### People who agreed to take part

116 of the eligible service users on the eight wards agreed to participate in the study. This constituted 60 % of all people eligible on the ward during the data collection period; the remaining service users were either too ill to be approached (*n* = 26) or did not want to participate (*n* = 49). The average age of participants was 39 years (SD 13) and 47 % (*n* = 54) were women (average age for women = 41 years). Seventeen (15 %) were married or had a partner. Sixty-four were white (56 %) with the remaining 50 (46 %; two individuals declined to answer) belonging to black or ethnic minority communities. Forty-three participants (38 %) had some further education, and 31 (27 %) were in employment. The average length of stay was 93 days (median 47) with a range of 5 days to 3 years and 3 months. Over half of the patients in the current study were diagnosed with psychotic (*n* = 50; 43 %) or bipolar (*n* = 20; 17 %) disorders, 13 % with mood disorders (*n* = 12; 10 %) and personality disorder (*n* = 13; 11 %), substance abuse (*n* = 6; 5 %) and other disorders (*n* = 13; 11 %). The remaining two individuals did not give consent for us to look at their medical records. The mean PANSS total score was 55.36 (SD 12.9) with that for the positive subscale being 12.41 (SD 4.1) and 12.33 (SD 4.5) on the negative subscale. Just under a third (*n* = 33) described delusions rated at least moderate severity (a rating of four or more on the PANSS).

### What activities did patients take part in?

Across all 8 wards, 116 participants took part in 436 group activities organised by ward staff over the previous 7 days, an average of 3.8 (SD 5.1) per participant. Eighteen per cent (*n* = 79) of all this activity involved formal therapy (e.g. art therapy, problems solving skills group), 32 % (*n* = 142) were attendance at community/daily planning meetings and 49 % (215) were classed as other activities (e.g. pottery group, bingo). Looked at from the perspective of individual participants, over a quarter (28 %; 32/115) did not report participating in any formal activity. Of the 83 who took part in at least one activity, 42 (51 %) attended formal therapy, 42 had attended at least one community meeting and 61 (73 %) were engaged in activities such as bingo, discussion groups and arts and crafts. Taken together participation in group activities occupied on average, only 3.5 h per week.

The numbers of activities attended by each participant varied considerably between wards, from an average of 14.8 (SD 8.1) to 1.4 (2.4). This variability in attendance between wards was statistically significant (*F* 9.45, 7; *p* < 0.001), but one ward, the female-only ward-in-the-community, had very high rates of attendance across all categories. If this ward is excluded as a sensitivity analysis then the discrepancy in activity between wards remains (*F* 2.688, 6; *p* = 0.02) but cannot be explained by whether the ward was male only (mean 2.7, SD 4.1) or female only (mean 3.9, SD 3.1).

In addition to group activities, participants reported a mean of 4.6 (SD 4.9) one-to-one sessions with mental health staff over the past week, with a range of 0–25. The majority of these were with nursing staff. Eighteen participants (16 %) reported that they had no meaningful contacts at all. The total amount of time spent in one-to-one sessions was a reported mean of 50.3 (SD 54.6) minutes per week and this differed between wards (mean 12.3, SD 8.1–2.1 SD 1.9, *F* 4.52, 7; *p* < 0.001). Again, there was a high frequency in the ward in the community and when this was removed in a sensitivity analysis the differences were no longer significant (mean 6.0 SD 3.4–2.1 SD 1.9).

Combining the time spent in group activity with that in one-to-one sessions gives an average of around 4.3 h in total contact time per week, less than an hour a day. There were no significant differences in the amount or type of activity or of one-to-one contacts between men and women, by ethnic group, age, diagnosis or length of stay on the ward.

### Do acute mental health problems interfere with use of activities?

Of those taking part in activities, participants with a diagnosis of a psychotic condition reported taking part in fewer group activities than those with other diagnoses, though this was not statistically significant (mean 2.9 SD 3.7 vs. mean 4.9 SD 6.7, Mann–Whitney U *p* = n.s.). They also reported significantly fewer one-to-one sessions with staff (mean 3.8 SD 4.2 vs. 5.8 SD 5.6, Mann–Whitney U *p* = 0.04). There was no statistically significant correlation between levels of participation in group activity and GAF, NOSIE or PANSS positive or negative symptoms. However, participants who reported a complete absence of participation in activity had significantly higher average PANSS negative symptom scores than those who reported attending at least one group in the week (14.3 SD 5.1 versus 11.6 SD 4.2, *T* 2.62, *df* = 99, *p* < 0.01). Individual negative symptoms that were associated with an absence of activity were emotional withdrawal, passive social withdrawal and difficulty with abstract thinking.

Fewer one-to-one contacts with staff was correlated with higher PANSS negative symptoms (*r* = −0.21, *p* = 0.03) and low GAF function scores (*r* = 0.2, *p* = 0.05). NOSIE scores, which were a nurse-based measure of inappropriate behaviour, were also associated with fewer one-to-one contacts (*r* = −0.25, *p* = 0.02).

When these analyses were run separately for men and women these findings were broadly replicated. PANSS negative symptoms were associated with lower rates of one-to-one contact and those scoring high on the NOSIE reported fewer one-to-one contacts with staff (men *r* = −0.32, *p* = 0.02, women *r* = −0.25, *p* = 0.05).

### What other variables are related to activities used?

VOICE total score reflecting perceptions of the quality of the ward was correlated with the frequency of one-to-one staff contact (*r* = −0.29, *p* = 0.002) and with the number of group activities attended (*r* = −0.24, *p* = 0.002). Four VOICE items ask specifically about satisfaction with ward activity, and all show significant negative correlations (i.e. reflecting dissatisfaction) with levels of activity (range *r* = −0.194, *p* = 0.039 to *r* = −0.270, *p* = 0.007) and one-to-one sessions: ‘staff are available to talk to when I need them’ ‘activities on the ward meet my needs’; ‘I find one-to-one time useful’ and ‘I find sharing experiences in a group useful’. The item ‘I find one-to-one time useful’ also has a negative correlation with number of contacts (*r* = −0.307, *p* = 001).

Tests for gender associations showed that high VOICE scores reflecting dissatisfaction with the quality of care on wards were associated in women with fewer one-to-one contacts (r = −0.39, *p* = 0.04) and lower participation in activities (*r* = −0.37, *p* = 0.05).

Formally detained service users did not differ from those who were in hospital voluntarily on any measure of activity or one-to-one contact. Longer stay patients (defined as those who had been continuously hospitalised for more than the median of 47 days) were found to have poorer levels of function [GAF-F mean 37.5 SD 6.8 vs. 42.7 SD 9.0, *T* 2.87, df 76, (mean difference 5.2) *p* < 0.01] but did not differ in terms of PANSS or NOISE scores or number of activities or contacts with staff.

The bivariate associations for the frequency of contacts were further explored through generalised estimating equations with total number of contacts treated as clustered by ward. Table 1 shows that four variables were significantly associated with contact frequency: total VOICE score representing greater dissatisfaction with care, total NOSIE score representing nurse observations, total PANSS negative symptom score and total GAF score. The associations remain significant when account is taken of age, gender and ethnicity though PANSS negative symptoms are no longer significant once mental health status (i.e. whether hospitalisation was voluntary or not ) is taken into account.

**Table 1:** Modelling service user—staff contact: associations with clinical function, nursing observation and patient satisfaction with care

	IRR	(95 % CI)	*p*	Adjusted for ethnicity, gender, age			Adjusted for ethnicity, gender, age and MHA status*		
				IRR	(95 % CI)	*p*	IRR	(95 % CI)	*p*
PANSS negative	0.96	(0.93, 0.98)	0.000	0.96	(0.93, 0.99)	0.021	0.97	(0.94, 1.00)	0.060
GAF total	0.98	(0.97, 0.99)	0.023	0.98	(0.97, 0.99)	0.003	0.98	(0.97, 0.99)	0.011
NOISIE total	0.97	(0.95, 0.99)	0.024	0.97	(0.95, 0.99)	0.015	0.97	(0.96, 0.99)	0.024
VOICE total	0.98	(0.97, 0.99)	0.000	0.98	(0.97, 0.98)	0.000	0.98	(0.97, 0.99)	0.000

* MHA status = whether compulsorily detained in hospital or not

## Discussion

A great deal of effort, time and money has gone into improving mental health services over the past 50 years and few would doubt the importance of activity and occupation in recovery. It would be expected, therefore, that of all the changes in inpatient settings, the delivery of activity both therapeutic and leisure would be most prominent.

The Three Hospitals Study [2] carried out in the 1960s assessed activity through reports from the head nurse and other staff, who observed what service users on the wards were doing. Patients in two hospitals spent around 4–5 h off the ward each day; most of this working, with approximately an hour with an occupational therapist with a further 2 h a day was spent on leisure activities. The third hospital was quite different with only around 1 h spent off the ward and 3 h in work or leisure activities. A clear relationship was found between levels of activity and the severity of specific symptoms. Activity and symptoms were associated so that time spent doing nothing was positively correlated with flatness of affect (*r* = 0.494), poverty of speech (*r* = 0.472) and social withdrawal (*r* = 0.634). In contrast, activity was not correlated with either incoherence of speech (*r* = 0.130) or delusions (*r* = −0.077).

In our study, we also observed a considerable variation between wards in the amount of activity and number of one-to-one contacts with staff and even after the sensitivity analysis there were still significant differences between wards. This variation did not seem to be explained by service user characteristics such as diagnosis, gender, ethnicity or length of stay. Considerably less time was spent in all forms of activity than in even the least active of the three hospitals 50 years ago at just over 3.5 h per week. Fully a quarter of patients reported no activities at all, this despite the much higher staff to patient ratio than in the 1960s. Curson et al. [16] replicated the Three Hospitals study, using the same methodology as Wing and Brown, comparing a single ward to the original three. As did we, they found that in 1990, service users were taking part in fewer activities than they did in the 1960s in spite of the high nurse to patient ratio.

Although mental health problems in our participants did not prove to be a barrier to taking part in activities, the explanation for the apparent dearth of activity lies in part with the severity of illness in the inpatient settings with the nurses immersed in immediate crisis management or dealing with the demands of paperwork and administrative tasks. Nurses in our study also reported gaps in activity due to staff shortages or absence so that the staff member skilled in running an activity was not available. This puts strain on the nurses who are on the ward, and bank staff may not have the knowledge to run the groups and neither do they know the patients as well. Furthermore, it is no longer common practice to involve service users in activities such as grounds keeping.

One of the reasons often given by staff for not providing or running groups on acute wards is that service users are too ill to appreciate or take part in structured activities. However, as with the earlier studies we found no significant association between activity and positive symptoms or general distress, and the association with negative symptoms was only significant for the relatively small number of patients who took part in no activity at all. Counts of group activity were correlated with the frequency of one-to-one staff contact which also was not correlated with positive symptoms and only weakly associated with negative symptoms.

Importantly, both activity and one-to-one sessions were associated with service users’ perceptions of the quality of care. In recent times, across all areas of health care, engaging stakeholders in their care has been seen as an important step in improving care. These findings demonstrate that even those on an acute ward can engage in and appreciate the activities provided and the meaningful time they can have with staff which is reflected in their perceptions of the ward. Moreover, this reiterates the importance of involving our clients in their care planning as their contributions and expertise have proven value.

## Limitations of the study

Our study is a snapshot of 1 week in the life of a small number of acute inpatient wards in one mental health Trust that serves a largely urban and inner city community and may not be representative of other Trusts. Furthermore, with a 40 % being either too ill to consent or refusing to participate, our sample may not be representative. Being a cross-sectional study, we only report associations: it is not possible to determine the causal direction of the association between rates of contact with staff and negative symptoms or dissatisfaction with services.

Since our measures were based on service user self-report, it is possible that taking part in therapeutic activities is underreported. We did not measure informal leisure activities between service users and although not measured directly, we did observe brief casual encounters between nurses and service users throughout the day and these everyday exchanges certainly contribute to the ward atmosphere and are normalising practices that mitigate institutionalisation.
